# Selenium in tea plant cultivation: bioavailability, uptake, metabolism, and physiological regulation

**DOI:** 10.3389/fpls.2025.1718992

**Published:** 2025-11-13

**Authors:** Lijin An, Yingxin Mao, Danjuan Huang, Yang Leng, Xun Chen

**Affiliations:** 1 Key Laboratory of Tea Resources Comprehensive Utilization, Ministry of Agriculture and Rural Affairs, Institute of Fruit and Tea, Hubei Academy of Agricultural Sciences, Wuhan, China; 2 National Agricultural Technology Extension and Service Center, Ministry of Agriculture and Rural Affairs, Beijing, China

**Keywords:** selenium, *Camellia sinensis*, abiotic stress, metabolic regulation, Se-enriched tea

## Abstract

Selenium (Se) is a trace element essential for human health. Incorporating Se into the tea (*Camellia sinensis* (L.) O. Kuntze) cultivation has emerged as a cropping technology in the area of agriculture and food sciences. The production of Se-enriched tea is greatly influenced by Se bioavailability in tea garden soil, which in turn influences the Se assimilation, absorption, and transportation of tea plants. Recent studies reported that Se can regulate tea plant growth by altering soil microbes, thereby improving soil fertility and enhancing tolerance to abiotic stressors such as pesticide toxicity, fluoride toxicity, and temperature extremes. Selenium can modulate the secondary metabolism of tea and design Se-rich functional components, which determine the quality of Se-enriched tea. This review seeks to provide theoretical bases for optimizing Se management in tea gardens and the cultivation of Se-enriched tea.

## 1 Introduction

Selenium (Se) is essential for humans; in higher plants, including tea, it is not essential but often acts as a beneficial element at low doses (Winther et al., 2020; Xia et al., 2021). Almost 15% of the world’s population is affected by Se deficiency, which leads to the risk of Keshan disease, thyroid dysfunction, and cardiovascular disease (Natasha et al., 2018). Therefore, developing safe and high-efficiency approaches and techniques to supplement Se intake is critical for the maintenance of human health (Li et al., 2023a). Selenium biofortification in tea plants (*Camellia sinensis* (L.) O. Kuntze) through foliar spraying has gained significant attention owing to its health benefits (**Table 1**; Wang et al., 2024). Tea plants can take up and transform inorganic Se into organic form (Ma et al., 2024; Wang et al., 2024). The tea plant predominantly assimilates inorganic selenite (SeO_3_²^-^) and selenate (SeO_4_²^-^) ions (Trippe and Pilon-Smits, 2021); SeO_3_²^-^ is absorbed through a phosphate transporter, while SeO_4_²^-^ is absorbed through a sulfate transporter. The absorbed inorganic Se will be transformed into organic forms via catalytic enzymes such as ATP sulfurylase and cysteine synthase (Natasha et al., 2018).

**Table 1:** Effects of Se application on growth, functional components, and stress tolerance of tea plants.

Cultivar/Area	Dose and form of Se	Treatment	Leaf Se content (DW)	Main effects	Reference
Shanghai	1.67 to 13.33 g L^-1^ Se-glucosamine	Foliar spraying	0.25–3.07 μg g^-1^	Improvement of photosynthesis and antioxidant system	Xu et al., 2024
Fuding-dabai	100 mg kg^-1^ SeNPs	Foliar spraying	180-fold higher than the control	Reduction of the phenol-to-amino acid ratio	Zhang et al., 2024a
Xinyang 10	100 mg L^-1^ Na_2_SeO_4_	Foliar spraying	1.62 μg g^-1^	Enhancement of glucose, tea polyphenol, catechin, and flavonoid contents	Sun et al., 2022
	100 mg L^-1^ Na_2_SeO_3_		1.65 μg g^-1^		
	100 mg L^-1^ Se yeast		2.06 μg g^-1^		
Longjing 43	10 mg L^-1^ SeNPs	Foliar spraying	10-fold higher than the control	Promotion of nitrogen availability and amino acid accumulation	Liu et al., 2025
Anhui	0.25, 0.5, and 1.0 mg L^-1^ Na_2_SeO_3_	Hydroponics	4.0- to 11.7-fold higher than the control	Mitigation of fluoride stress and content	Niu et al., 2020
Zhongcha 108	2.5, 5, 10, and 20 mg kg^-1^ SeNPs	Foliar spraying	NA	Mitigation of pesticide stress and enhancement of carotenoid, tea polyphenol, and catechin contents	Li et al., 2021
Longjing 43	2 mg L^-1^ Na_2_SeO_3_	Foliar spraying	NA	Enhancement of cold tolerance and the contents of polyphenols, sugars, and amino acids.	Liu et al., 2021
Bixiangzao	3.75, 5.0, 7.5, and 15 mg L^-1^ SeNPs	Foliar spraying	0.08–0.15 μg g^-1^	Reduction of bitterness and astringency with enhancement of umami and sweetness in tea	Huang et al., 2023
Fuding-dabai	5, 10, and 50 mg L^-1^ SeNPs	Foliar spraying	NA	Promotion of 100-bud weight and contents of theanine, total catechin, and caffeine	Zhang et al., 2024b
Fuding-dabai	60 mg L^-1^ Se-glycine	Foliar spraying	4.08–6.89 μg g^-1^	Promotion of tea polyphenols accumulation	Li et al., 2023b
Fuding-dabai	200 mg L^-1^ Se-glycine	Soil fertilization	1.31–2.39 μg g^-1^	Promotion of tea polyphenols accumulation	Li et al., 2023b
Jiangsu	150 mg L^-1^ Organic Se fertilizer	Foliar spraying	5.03 μg g^-1^	Enhancement of vitamin C content, sweetness, and aroma of tea	Huang et al., 2005

NA, not analyzed; DW, dry matter.

The application of Se to tea gardens enhances soil health and promotes tea plant growth (Li et al., 2021; Zhang et al., 2024a). The activities of soil microorganisms would be enhanced by Se and beneficial microbial communities enhancing nitrogen and sulfur cycling would be fostered by Se as well, thereby improving the structure of soil nutrient supply and soil fertility (Guo et al., 2024). In addition, Se can activate the antioxidant system of tea plants, such as increasing the activity of glutathione peroxidase, and also enhancing the structural stability of their cell membranes (Xu et al., 2024). These can help tea plants against abiotic stresses, like pesticides, fluoride toxicity, and temperature extremes, thereby improving their quality (Niu et al., 2020; Liu et al., 2021). Moreover, Se affects the secondary metabolism of tea and changes the content of functional ingredients, including polyphenols, amino acids, and vitamins, thus modulating the taste and nutritional benefit of the tea (**Table 1**; Ye et al., 2023). Se-enriched tea is not only a food source to supplement Se, but also has stronger antioxidant, anti-inflammatory, and other biological activities than conventional tea (Gu et al., 2020; Ma et al., 2024).

Current studies initially investigated the Se bioavailability in tea garden soils and uncovered partial Se transformation mechanisms of tea plants (Ren et al., 2022; Guo et al., 2024; Wang et al., 2024). However, the key factors influencing Se bioavailability in soil, the regulatory network governing Se translocation within tea plants, and the precise regulatory patterns of Se on critical secondary metabolic pathways in tea plants require further investigation. Hence, we systematically summarize the regulatory mechanisms of key factors influencing Se bioavailability in tea garden soils and Se metabolism mechanisms of tea plants. We also discuss regulatory roles of Se on tea plant growth, stress tolerance, and functional components. The purpose is to provide theoretical support and technical references for the sustainable cultivation of the Se-enriched tea.

## 2 Bioavailability of Se in tea garden soils

**Figure 1:**
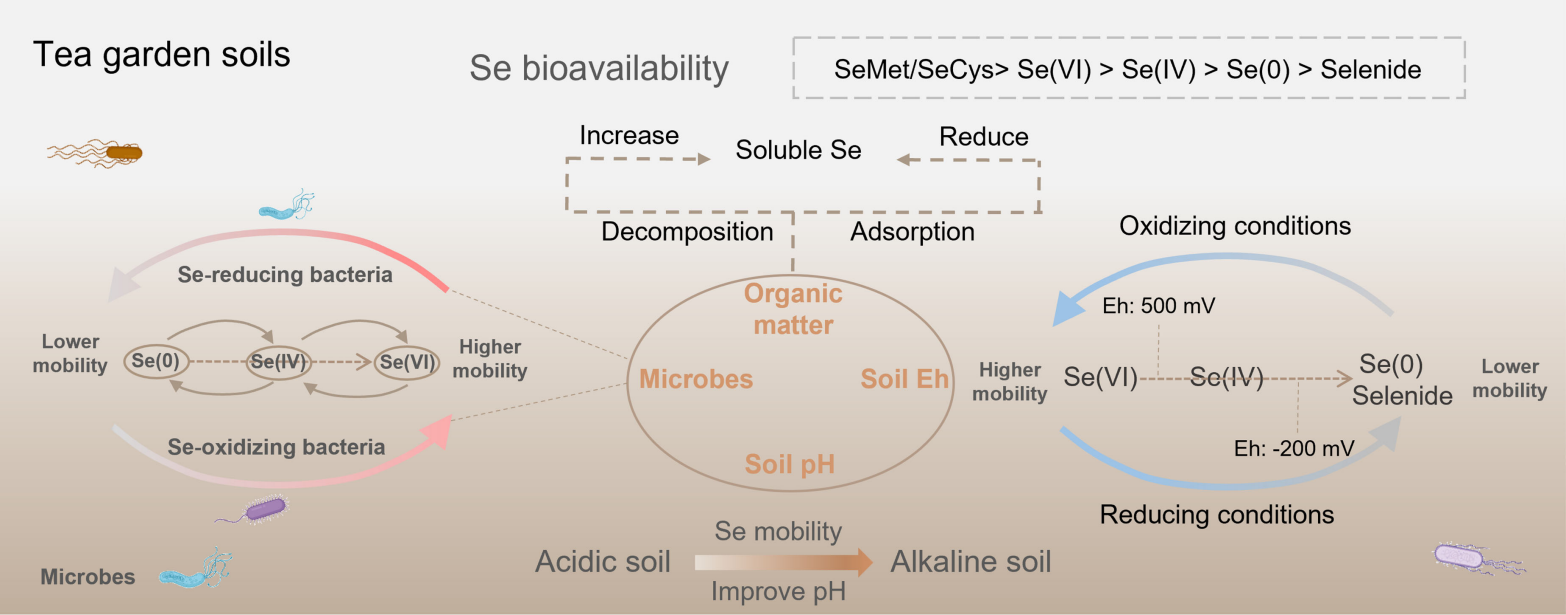
Se bioavailability in tea garden soils is regulated by many factors, including Se speciation, the soil physicochemical properties, and the rhizosphere microbial community.

Selenium bioavailability in tea garden soils is influenced by many factors, including Se speciation, the physicochemical properties of the soil, and the microorganisms in the rhizosphere (**Figure 1**; Natasha et al., 2018; Wu et al., 2024). These factors directly determine the Se uptake efficiency of tea plants and thereby sequentially influence the Se content in tea leaves and the quality of Se-enriched tea.

### 2.1 Se speciation

Selenium speciation in soil can be categorized into inorganic and organic forms. Inorganic Se consists of selenide (Se[−II]), elemental selenium (Se[0]), selenite (Se[IV]), and selenate (Se[VI]) (Tolu et al., 2011). Organic Se comprises selenomethionine (SeMet), selenocysteine (SeCys), and methylselenocysteine (MeSeCys) (Weng et al., 2011; Natasha et al., 2018). Elemental Se and selenide exhibit poor water solubility and low bioavailability, making them hardly absorbable by plants. By contrast, due to their high water solubility, Se(VI) and Se(IV) can be efficiently taken up by plant roots (Peng et al., 2017; Wang et al., 2017). Selenoamino acids such as SeMet and SeCys possess higher bioavailability than inorganic forms and are thereby easily absorbed and assimilated by plants (Kikkert and Berkelaar, 2013). Inorganic Se is widely recognized as the predominant Se form in agricultural soils (Guo et al., 2022). However, organic Se content of many Se-rich farmland soils is reported as high as 56-81% (Qin et al., 2017). Due to the unique biogeochemical characteristics of tea garden ecosystems, more research is required to investigate the ratio of inorganic Se to organic Se in tea garden soils with abundant leaves and humus.

### 2.2 Physicochemical properties of soil

Physicochemical soil parameters (e.g., pH, organic matter, redox potential [Eh]) directly affect Se bioavailability (Natasha et al., 2018; Guo et al., 2022). Soil pH affects the chemical forms of Se, influencing its bioavailability (Lyu et al., 2021). Under acidic conditions, SeO_3_²^-^, which is less bioavailable due to its strong adsorption to soil minerals, is the predominant Se species (Peak, 2006). Under alkaline soils, SeO_4_²^-^ is the most common form, with greater solubility and bioavailability (Gupta and Gupta, 2017). Although tea plant grows well in acidic soils (pH 4.0-5.5) with high aluminum activity (Wang et al., 2024), acidic tea garden soils can release Al³^+^, which reacts with SeO_3_²^-^ to form the insoluble precipitate Al_2_(SeO_3_)_3_, thereby reducing Se bioavailability (Yang et al., 2023). Some tea plantations suffer from severe soil acidification, with pH levels dropping below 4.0. This condition inhibits tea plant growth and reduces Se mobility in the soil (Lyu et al., 2021; Wu et al., 2024). Therefore, agronomic management practices that ameliorate soil acidity in these plantations can also improve Se bioavailability (Tian et al., 2016). In addition, soil available Se is positively correlated with soil Eh (Li et al., 2010). Se oxyanions will be reduced to Se(0) and selenide under a waterlogged reductive environment with Eh < -200 mV, thereby decreasing bioavailability (Masscheleyn et al., 1991). When soil Eh ranges from 0 to 200 mV, Se mainly exists in the form of Se(IV). However, Se predominantly exists as Se(VI) with the highest bioavailability under an oxidative environment with Eh > 500 mV (Nakamaru and Altansuvd, 2014; Natasha et al., 2018). Organic matter of tea garden soils, which is mainly derived from tea plant litter and pruning residues, exerts a dual-directional effect on Se bioavailability (Qin et al., 2017). Organic matter produces organic acids through microbial mineralization, which promotes the desorption of Se from mineral surfaces. Meanwhile, macromolecular selenoproteins in organic matter are mineralized into inorganic Se, thereby enhancing Se bioavailability (Wu et al., 2024). However, functional groups in organic matter, such as carboxyl groups and phenolic hydroxyl groups, can adsorb Se oxyanions through complexation, forming organic-bound Se and thereby reducing Se bioavailability (Qin et al., 2012; Li et al., 2017).

### 2.3 Roles of soil microbes

Microorganisms are important members of the soil-plant system (Eswayah et al., 2016). They are essential in modulating Se bioavailability by participating in biotransformation processes, including Se oxidation, reduction, and dissolution (Wells and Stolz, 2020) (**Figure 1**). Some studies found that Se-oxidizing bacteria oxidize organic and elemental Se into more mobile Se oxyions, which promoted Se absorption by plants (Losi and Frankenberger, 1998; Luo et al., 2022). For example, applying Se-oxidizing *Agrobacterium* sp. T3F4 enhanced Se bioavailability and Se absorption by *Brassica rapa* L (Zhu et al., 2021). It is highly necessary to investigate the effects of Se-oxidizing bacteria on the Se bioavailability in tea garden soils and Se accumulation of tea plants in future studies. Conversely, numerous Se-reducing microbes have been confirmed to convert Se(VI) and Se(IV) into insoluble elemental Se, which substantially diminishes its bioavailability (Eswayah et al., 2016). In addition, bacteria capable of solubilizing Se minerals show considerable potential to enhance Se bioavailability. *Caulobacter vibrioides* T5M6, isolated from Se ore in Enshi, China, exhibited a significant capacity for the solubilization of Se minerals and produced bioavailable Se(IV) (Wang et al., 2016). Several studies confirmed the crucial function of soil microbes in promoting Se absorption by tea plants. Xu et al. (2020) showed that inoculating the tea plant with *Herbaspirillum* sp. WT00C increased the organic Se concentration of tea leaves. Applying *Bacillus amyloliquefaciens* promoted the expression of the phosphate transporter gene *CsPHT1;2b*—which is involved in Se(IV) absorption by tea plant—increasing Se concentration of tea leaves by 57.0% (Li et al., 2025). These findings indicate that the functions performed by soil microorganisms are diverse and critical in regulating Se biogeochemical cycling in tea garden soils (**Figure 1**). This provides essential information for developing microbial-based strategies for Se-enriched tea cultivation.

## 3 Uptake, translocation and assimilation of Se by tea plants

Tea plants are not hyperaccumulators of selenium but possess relatively high selenium uptake and accumulation capacity. Selenium is not an essential element for tea plants, so they have not evolved specific selenium uptake and assimilation pathways. Instead, they primarily rely on sulfur assimilation pathways to accumulate organic selenium. The Se content of tea leaves grown in Se-rich soil can reach the Se-enriched tea standard of 0.25 to 4.00 mg/kg (Ren et al., 2022; Wu et al., 2024). Therefore, studying the mechanisms of Se absorption, translocation, and transformation in the tea plant is critical for improving the tea quality, especially in Se-deficient areas.

### 3.1 Se uptake in roots

**Figure 2:**
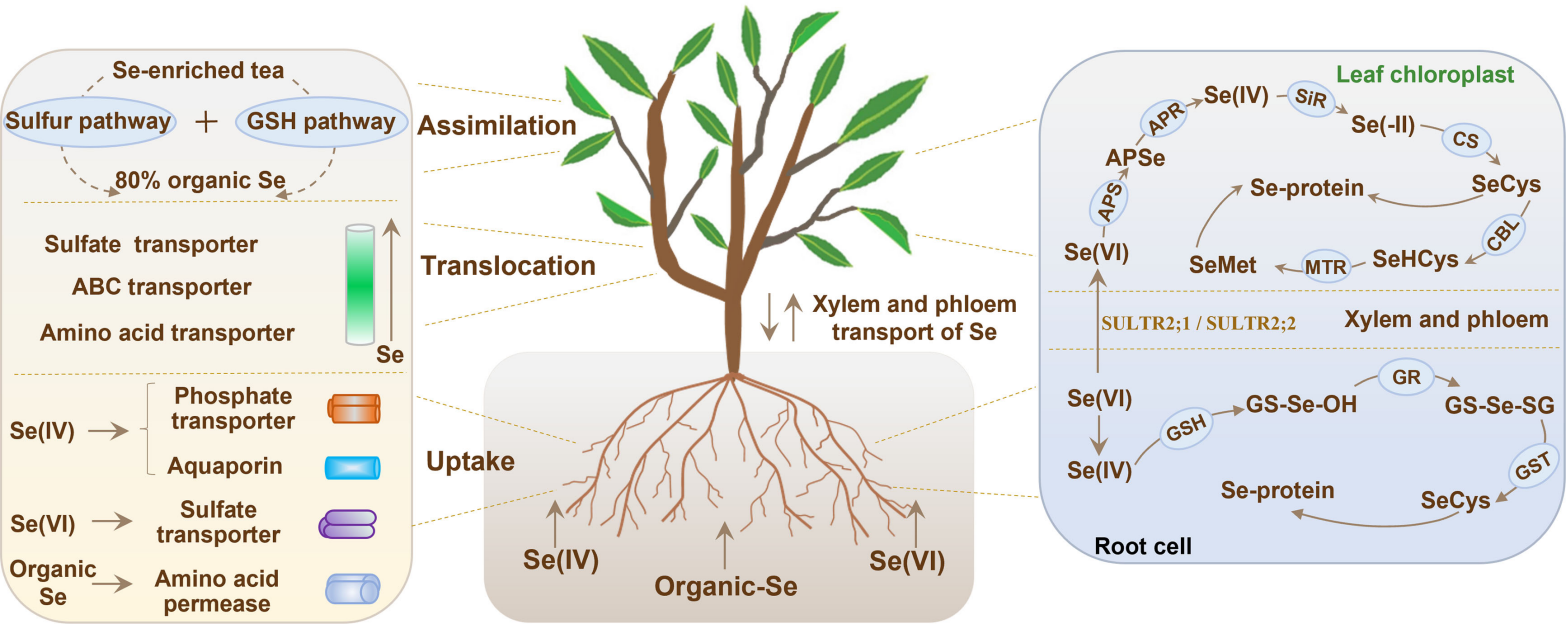
Mechanism of Se uptake, translocation and assimilation within tea plants. GSH, glutathione; GR, glutathione reductase; GST, glutathione S-transferase; SULTR2;1, sulfate transporter 2;1; SULTR2;2, sulfate transporter 2;2; APS, adenosine triphosphate sulfurylase; APSe, adenosine 5’-phosphoselenate; APR, adenosine 5′-phosphosulfate reductase; SiR, sulfite reductase; CS, cysteine synthase; CBL, cystathionine-β-lyase; MTR, methionine synthase.

The roots of the tea plant primarily rely on sulfur, phosphorus, and amino acid transporters to uptake soluble Se(IV), Se(VI), and selenoamino acids from soil (**Figure 2**; Trippe and Pilon-Smits, 2021). Se(VI) uptake by plants primarily relies on sulfate (SO_4_²^-^) uptake channels because of their chemical similarity (White, 2018). Research has shown that Se(VI) is absorbed by tea plants through members of the sulfate transporter (SULTR) family. The expression of *SULTR1;1* and *SULTR2;1* in tea plant roots is significantly upregulated when treated with Se(VI), which confirms the important role of these transporters in Se(VI) uptake (Ren et al., 2022). In tea cultivars with high Se accumulation capacity, the expression of *SULTR1;2* and *SULTR3;4* was increased by treatment of Se(VI), suggesting that these two transporters may be responsible for the efficient transfer of Se from roots to above-ground parts (Zheng et al., 2023).

Se(IV) uptake is mainly mediated by phosphate (PO_4_³^-^) transporters (PHTs) because of their structural similarity (Wang et al., 2024). Phosphate transporter genes such as PHT3;1a, PHT1;3b, and PHT1;8 in tea plants are induced under Se(IV) treatment (Cao et al., 2021). Besides PHTs, transport of Se(IV) across cell membranes also involves aquaporins. Se(IV) treatment leads to the upregulation of a gene called NIP2;1, suggesting that this gene may act as a nonspecific channel for the uptake of Se(IV) (Ren et al., 2022). Several transporters including ATP-binding cassette (ABC) transporter, glutathione S-transferases (GSTs), and nitrate transporter 1 (NRT1), were found to alter their expression under Se(IV) treatment, implying that Se(IV) uptake is a combined action of several transporters (Zheng et al., 2023).

Se(IV) uptake of plants is also significantly influenced by soil pH. In acidic soil (pH 5.0), Se predominantly exists in the form of HSeO_3_^-^. Conversely, under alkaline soil (pH > 7.0), SeO_3_²^-^ is the major form. Phosphate transporters have a greater affinity for HSeO_3_^-^ than for SeO_3_²^-^ (Zhang et al., 2010), meaning that tea plants growing in acidic soils can absorb Se(IV) better than those growing in alkaline soils (Wang et al., 2024). Tea plants growing in acidic soils still show a high capacity for Se enrichment, even though Se is much less mobile in acidic soils than in alkaline soils. Additionally, plants can absorb selenoamino acids such as SeMet and SeCys through amino acid transporters in their roots, including amino acid permease 1 (AAP1) and lysine-histidine transporter 1 (LHT1) (**Figure 2**). Several studies have demonstrated that the uptake efficiency of selenoamino acids by plants exceeds that of inorganic Se. Kikkert and Berkelaar (2013) reported that the SeMet uptake efficiency by wheat was 100 times greater than that of Se(VI). However, further investigations are needed to elucidate the uptake efficiency and specific mechanisms of organic Se in tea plants.

### 3.2 Se translocation in shoot

Selenium is transferred to the leaves of tea plants by xylem and phloem for accumulation and metabolism (Natasha et al., 2018) (**Figure 2**). Notably, there are significant differences in Se(IV) and Se(VI) transport pathways in tea plants. Se(IV) will be converted into organic Se and selenoglutathione (GSSeSG) (Somagattu et al., 2024). However, organic Se is difficult to be translocated via the xylem, which leads to the majority of Se(IV) being sequestrated in the root cell vacuoles (Gupta and Gupta, 2017). In contrast, Se(VI) is more easily loaded into xylem vessels via the sulfate transporters SULTR2;1 and SULTR2;2, and then transported to and accumulated in the leaves along with the plant’s transpiration stream (White, 2018; Ren et al., 2022). Selenium transport in the xylem of plants is also under the control of plant hormones. For example, jasmonic acid (JA) and ethylene can stimulate *SULTR2;1* expression, leading to the enhancement of Se(VI) xylem loading (Cao et al., 2018; Zheng et al., 2023). Further, organic Se translocation and redistribution to above-ground organs is largely a function of the phloem tissues. Selenoamino acids are transported through the companion cell-sieve tube complex in phloem of rice, relying on amino acid transporters of the AAP family and ABC transporters of the ABCG family (Wang et al., 2024). In tea plants, two genes called *CsAAP7* and *CsABCG11* were overexpressed quite a lot when they were put under Se exposure. This indicates that these two genes may help transport organic Se (Cao et al., 2021; Ren et al., 2022). Phloem translocation of organic Se in tea plants needs more in-depth investigation for understanding the underlying mechanism involved in the process.

### 3.3 Se assimilation in roots and leaves

Over 80% inorganic Se will be converted into organic form by tea plants (**Figure 2**), which is more available for humans compared to inorganic Se (Chen et al., 2015). Se(IV) is mainly taken up and assimilated in root cells, whereas Se(VI) is mainly assimilated in leaf chloroplasts (Wang et al., 2024). In root systems, glutathione (GSH) first reacts with Se(IV) to form GS-Se-OH and is further reduced to form GS-Se-SG by glutathione reductase (GR) (Cao et al., 2018; Natasha et al., 2018). Subsequently, GS-Se-SG is transformed into SeCys through the catalytic activity of glutathione S-transferase (GST) and SeCys is then incorporated into the synthesis of selenoproteins (Gupta and Gupta, 2017).

Unlike Se(IV), Se(VI) is converted into adenosine 5′-phosphoselenate (APSe) in chloroplasts by the activity of adenosine triphosphate sulfurylase (APS) (Sors et al., 2005; Ren et al., 2022). APSe is then reduced to Se(IV) by APS reductase (Somagattu et al., 2024). Sulfite reductase further reduces the produced Se(IV) to Se(−II), which reacts with O-acetylserine to generate SeCys by cysteine synthase. SeCys is then transformed into selenohomocysteine via cystathionine-β-lyase, and eventually converted to SeMet through the catalysis of methionine synthase (Lima et al., 2018; Trippe and Pilon-Smits, 2021). Notably, SeCys can be methylated by selenocysteine methyltransferase to synthesize methylselenocysteine (MeSeCys) in tea plants (Trippe and Pilon-Smits, 2021; Wang et al., 2024). Excessive production of MeSeCys is sequestrated in vacuoles, functioning as a crucial mechanism that tea plants use to mitigate Se toxicity.

## 4 Se improves the growth and stress tolerance of tea plants

### 4.1 Se promotes tea plant growth

**Figure 3:**
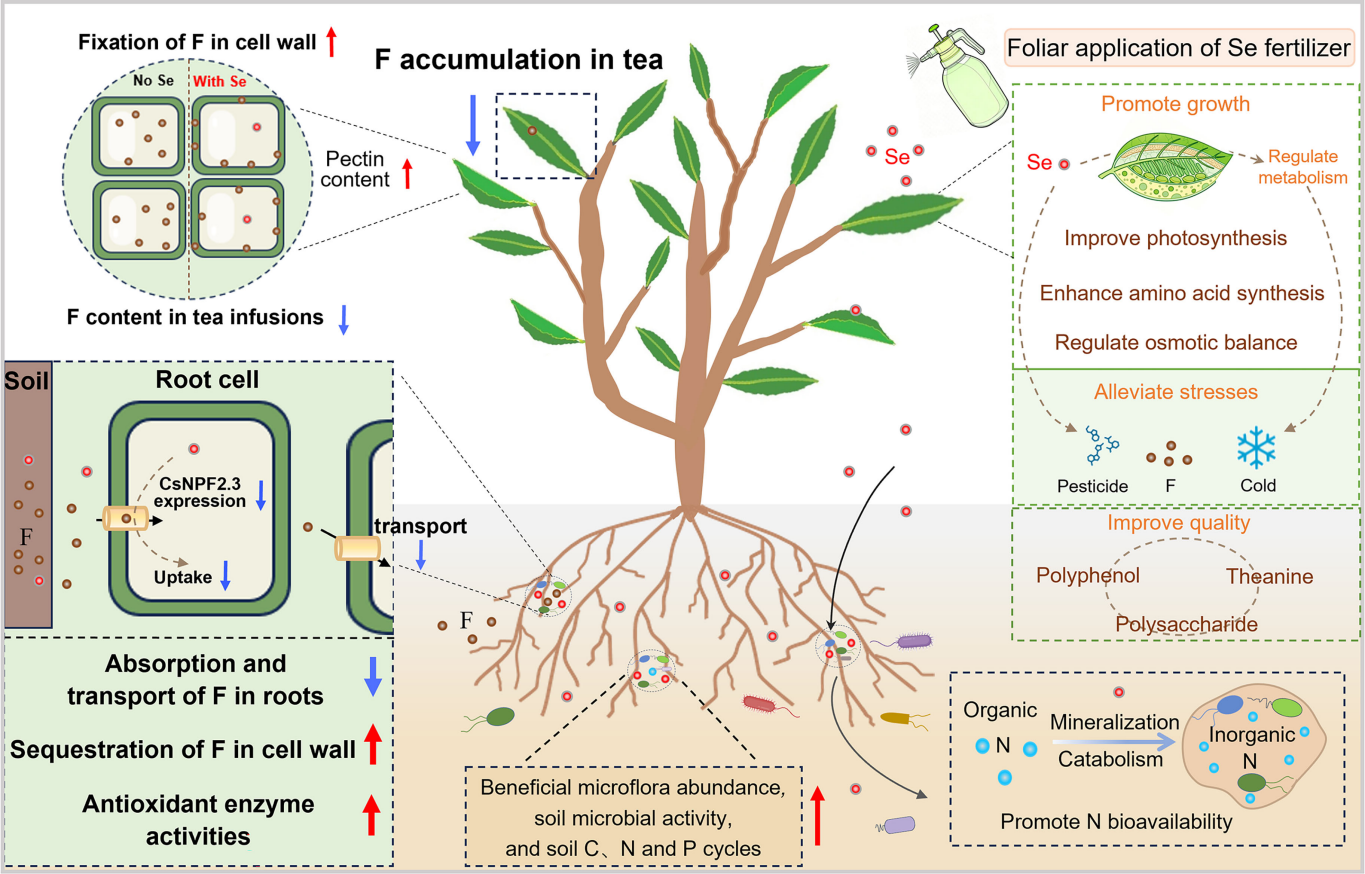
The mechanism of Se in enhancing tea plant growth and abiotic stress resistance.

Selenium plays a vital function in tea plant growth by regulating their physiological metabolism and nutrient acquisition from soil, as shown in **Figure 3** (Wang et al., 2024; Liu et al., 2025). Hu et al. (2003) showed that applying 60 mg L^-1^ Se fertilizer increased theanine content in early-spring tea and nearly doubled tea yield. Zhang et al. (2024a) demonstrated that applying nanoparticles (SeNPs) to tea plants significantly enhanced their antioxidant capacity by upregulating the activities of antioxidant enzymes such as catalase (CAT) and superoxide dismutase (SOD). The application of SeNPs also improved the soil bacterial community of tea gardens, but did not alter bacterial diversity or dominant taxa. These promotions contributed by SeNPs, which decreased the phenol-to-amino acid ratio, resulted in improved taste and flavor of tea (Zhang et al., 2024a).

Sun et al. (2022) further revealed that spraying Se promoted tea plant growth and promoted the absorption of nitrogen, phosphorus, and potassium in leaves. Specifically, Se supplementation upregulated the expression of amino acid synthesis-related genes (*CsGS, CsGOGAT*, and *CsHMGR*), which in turn increased amino acids, glucose, and tea polyphenols contents of tea. Beyond regulating physiological metabolism, Se has been demonstrated to enhance nitrogen absorption by plants through regulating soil nitrogen cycling. For legumes, Se application enhances the abundances of nitrogen-fixation genes (*nifH*) and nitrification genes (*amoA* and *nxrA*) by increasing the abundances of nitrogen-fixing and nitrifying bacteria in the legume rhizosphere, thereby increasing the content of bioavailable soil nitrogen and promoting the growth of legumes (Lei et al., 2022). For tea gardens specifically, application of SeNP fertilizer enhanced nitrogen bioavailability of tea garden soil by promoting the mineralization of soil organic nitrogen and reducing soil microbial denitrification, thereby improving tea quality and tea plant growth (Liu et al., 2025) (**Figure 3**). Se has been used as a fertilizer that enhances tea yield and quality, highlighting its significant value in the cultivation of high-quality tea.

Numerous studies have shown that foliar Se application is the primary agronomic measure for cultivating Se-rich tea, and its efficacy is significantly superior to that of root Se application (Zhang et al., 2024a; Liu et al., 2025). For instance, the Se content in tea leaves treated with a 60 mg L^-1^ Se-glycine (Se-Gly) foliar spray is nearly 5 times higher than that in tea leaves where 200 mg L^-1^ Se-Gly is applied to the rhizosphere soil (Li et al., 2023b). Selenium applied via foliar spraying can be directly and efficiently absorbed by tea leaves, which then rapidly converts it into organic Se. Notably, Se has a concentration-dependent dual effect on plant growth (Natasha et al., 2018). An appropriate dose of Se can improve antioxidant capacity and promote plant growth. Conversely, excess Se may cause oxidative stress, interfere with protein synthesis, and even be toxic to plants (Trippe and Pilon-Smits, 2021). However, because the assimilation of Se by tea plants is influenced by numerous factors, such as the form of Se and tea plant variety, a clear concentration threshold for Se in tea plants has not been established.

### 4.2 Se improves the tolerance of tea plants to abiotic stresses

A number of studies reported that Se can enhance tea plant tolerance to abiotic stresses such as fluoride, pesticides, and low temperatures by regulating the tea plants’ antioxidant systems and physiological metabolism (**Figure 3**). The tea plant is a fluoride hyperaccumulator, and reducing the fluoride content of tea is critical for ensuring the safety of tea consumption. Several studies demonstrated that applying Se reduces both fluoride accumulation in tea and the water-soluble fluoride proportion in tea infusions. Niu et al. (2020) showed that in a hydroponic system, applying 1.0 mg L^-1^ Se(IV) substantially decreased fluoride concentration of tea leaves by 34.7% while enhancing fluoride concentration of tea plant roots by 112%. Se(IV) enhanced the antioxidant enzyme activities and alleviated oxidative stress damage caused by fluoride in tea plants. Additionally, Se(IV) inhibited the abnormal accumulation of elements such as iron (Fe), calcium (Ca), and aluminum (Al) caused by fluoride exposure. These findings indicate that Se mitigates fluoride stress in tea plants by regulating elemental balance and the antioxidant system (Niu et al., 2020). Further studies examined the effect of Se in mitigating fluoride uptake through foliar spraying in a tea plantation. Spraying sodium selenite (Na_2_SeO_3_), selenocarbohydrate, SeMet, and nano-selenium (nano-Se) reduced the fluoride concentration of tea leaves in the range of 10.17-45.99% (Niu et al., 2023). Se promotes the content of pectin in cell walls by enhancing the activity of pectin methylesterase, resulting in the demethylation of pectin and sequestration of fluoride in cell walls (Niu et al., 2023) (**Figure 3**). Recently, Niu et al. (2025) uncovered the mechanism by which Se regulates fluoride absorption of tea plants. Transcriptomic analysis showed that applying Se significantly decreased the expression of transporter *CsNPF2.3*, which is expressed in root epidermal cells and xylem parenchyma cells. Functional validation via tea plant hairy root transformation experiments further confirmed that CsNPF2.3 acts as a key transporter mediating fluoride transport (Niu et al., 2025). Collectively, these findings demonstrate that Se application reduces fluoride translocation from roots to leaves by inhibiting the expression of the CsNPF2.3 transporter, thereby decreasing fluoride accumulation in tea leaves (**Figure 3**).

As another prevalent form of abiotic stress, pesticides will disturb the antioxidant system of tea plants and decrease the yield of tea. Applying 10 mg L^-1^ nano-Se improved ROS scavenging capacity in tea plants through increasing antioxidant enzyme (SOD, POD, and CAT) and ASA–GSH cycle, which could significantly lower malondialdehyde (MDA) and superoxide anion (O_2_^-^) levels (Li et al., 2021). Spraying nano-Se stimulated the production of amino acids like glutamate, proline, and arginine through modification of the glutamine-glutamate cycle, thereby enhancing the quality of tea under the stress of pesticides (Li et al., 2021). Yu et al. (2024) revealed that applying 2.5 mg L^-1^ nano-Se alleviated the inhibition of glutamine synthetase caused by glufosinate, thus promoting theanine content and improving tea flavor. In addition, Se has been shown to increase cold resistance of tea plants and tea quality under low temperature stress by regulating the physiology and metabolism of tea plants. Liu et al. (2021) showed that spraying 2 mg L^-1^ Na_2_SeO_3_ promoted the photosynthetic rate of Longjing 43 at a low temperature of 4°C. This treatment caused an increase in SOD and POD activities that mitigated oxidative damage caused by low temperature. Selenium also promoted the accumulation of soluble sugars and proline, further increasing the osmotic adjustment ability of tea plants (Liu et al., 2021).

## 5 Se modulates secondary metabolites in tea plants

The secondary metabolites of the tea plant not only serve as key defense substances for adapting to environmental stress, but are also the main functional components of tea (**Table 1**; Jiang and Wen, 2025). Selenium has been reported to regulate the synthesis of secondary metabolites of tea, including tea polyphenols, theanine, alkaloids, polysaccharides, and volatile compounds (Huang et al., 2005, 2023).

### 5.1 Regulation of tea polyphenols

Tea polyphenols are important functional substances in tea leaves, exhibiting antioxidant, anti-inflammatory, and anticancer properties (Ye et al., 2023). The application of Se was proven to regulate tea polyphenol synthesis, affecting the quality and nutritional components of tea. Huang et al. (2023) showed that spraying 15 mg L^-1^ nano-Se significantly reduced total tea polyphenols and catechins in summer and autumn tea. Specifically, a reduction in the key substances of epigallocatechin gallate considerably reduces the bitterness and astringency of infused tea. Application of nano-Se significantly downregulated multiple key genes in the phenylpropanoid biosynthesis and flavonoid biosynthesis pathways—such as phenylalanine ammonia-lyase and chalcone synthase—reducing the synthesis and accumulation of tea polyphenols.

However, applying Se does not consistently lead to a decrease in tea-polyphenol content. The regulatory effect of Se on tea polyphenols is closely associated with the growing season, specific tea variety, and application dose. Zhang et al. (2024b), for instance, showed that applying 10 mg L^-1^ nano-Se improved total catechins and EGCG in the Fuding Dabai tea cultivar during the spring season by increasing the expression of key genes involved in catechin synthesis, such as *CsPAL*, *CsC4H*, and *CsCHI*. Xu et al. (2024) demonstrated that spraying 1.67 g L^-1^ glucosamine selenium (GlcN-Se) promoted the catechin content in tea leaves by 41.78%, whereas application of 13.33 g L^-1^ GlcN-Se decreased it by 22.53%, indicating that Se exerts a dose effect on the regulation of catechin accumulation. Se application can also control the health-promoting characteristics and flavor of tea by regulating the composition of catechins (Ye et al., 2023). Se content had a strong positive correlation with galloylated catechins but a negative correlation with non-galloylated catechins. More research is needed to elucidate the Se metabolic mechanism in regulating these two types of catechins.

### 5.2 Regulation of theanine

Theanine is the core compound determining the flavor and freshness of tea and has multiple physiological functions, such as antioxidant and immunity regulation (Ye et al., 2023; Zhang et al., 2024b). Yet, summer-autumn tea’s theanine content is always low, which has long been a key problem that greatly damages its flavor and economic value (Jiang and Wen, 2025). Many studies reported that applying Se improved the flavor of summer-autumn tea, mainly by improving theanine levels. Huang et al. (2023) revealed that spraying nano-Se increased theanine content of summer tea by raising the expression of some key theanine biosynthetic enzymes, as GDH and GS/GOGAT. Moreover, treatment with 10 mg L^-1^ nano-Se improved amino acid biosynthetic inhibition caused by pesticides (Li et al., 2021). In this case, the theanine, glutamate, and aspartate contents increased by 16.4%, 55.4% and 45.5%, respectively. Because of low nitrogen metabolic activity, summer-autumn tea usually has a lower theanine content than spring tea. Hence, applying Se on stimulating the activity of the GS-GOGAT cycle is more efficient in autumn tea than in spring tea (Huang et al., 2023), thus facilitating more effective accumulation of theanine. Xu et al. (2024) indicated that theanine content in tea improved and then decreased with increased dosage of GlcN-Se. The Se regulatory role on tea theanine is associated with Se dosage, according to this finding. Additionally, applying GlcN-Se promoted umami amino acid and decreased the bitter amino acid content, thus promoting the flavor and quality of tea (Xu et al., 2024). The theanine content in tea leaves demonstrated an inverted-U-shaped relationship with increasing dosages of GlcN-Se, indicating that the regulatory effect of Se on theanine in tea leaves is closely associated with the Se dosage.

### 5.3 Regulation of polysaccharides

Tea polysaccharides are water-soluble biomacromolecules with immune-modulatory, hypoglycemic, and anti-tumor effects. According to Xu et al. (2024), the application of 13.33 g L^-1^ GlcN-Se improved the soluble sugar content of tea by 42.21%, increasing polysaccharide biosynthesis and accumulation. Applying 10 mg L^-1^ nano-Se promoted the net photosynthetic rate of tea plants by 48.7% and increased soluble sugar content by 30.3%, promoting polysaccharides biosynthesis and enhancing the sweetness of tea infusions (Zhang et al., 2024b). Selenium and polysaccharides in tea leaves can form a covalent conjugation to generate Se-containing tea polysaccharides (Se-TPs), further improving antioxidative activity and prebiotic effect of Se-enriched tea (Huang et al., 2023; Wang et al., 2024). Wang et al. (2015) showed that Se-TPs exhibited stronger scavenging activity against superoxide anion radicals than ordinary tea polysaccharides. In addition, two novel Se-TPs isolated from Se-enriched tea enhanced the antioxidant capacity of tea and effectively mitigated H_2_O_2_-induced DNA damage (Gu et al., 2020). It should be noted that Se-TPs from naturally Se-enriched tea are more stable than those of artificially Se-enriched tea (Zhu et al., 2019), so enhancing Se bioavailability in tea garden soil is an ideal approach for Se biofortification. In addition to possessing antioxidant and prebiotics, Se-TPs can also inhibit the proliferation of tumor cells, disrupting the tumor cell’s proliferation cycle and the activation of the mitochondrial apoptotic pathway (He et al., 2013; Wang et al., 2024). However, existing studies have been based on cellular and animal models, and there is no clinical trial data in humans. Future research will need to verify the effectiveness and safety of Se-TPs in preventing and treating human tumors.

### 5.4 Regulation of alkaloids

The alkaloids in tea mainly consist of purine derivatives such as caffeine, theobromine, and theophylline. They exhibit diverse physiological effects, including central stimulant activity, antioxidant activity, and metabolic regulatory activity, and also serve as the primary source of bitterness in tea. Selenium content is significantly correlated with caffeine and theobromine content (Ye et al., 2023). Zhang et al. (2024b) showed that applying 10 mg L^-1^ nano-Se improved the caffeine concentration of Fuding Dabai tea by 30.9%. Mechanistically, Se treatment significantly increases aspartate content, the initial precursor for caffeine, potentially providing sufficient substrates for caffeine biosynthesis (Xiang et al., 2022; Ye et al., 2023).

Notably, exogenous Se application does not always enhance alkaloid content of tea, as the Se regulatory role on alkaloids is closely associated with tea-growing seasons. Xu et al. (2024) showed that applying 13.33 g L^-1^ GlcN-Se reduced the caffeine content of summer tea by 28.15%. Spraying nano-Se with a dose of 5–15 mg L^-1^ was similarly found to significantly reduce caffeine content in tea leaves of summer tea (Huang et al., 2023). Transcriptomic analyses showed that applying nano-Se significantly reduced the expression of key caffeine biosynthesis genes, including adenine phosphoribosyltransferase and adenosine monophosphate deaminase, thereby reducing caffeine accumulation in summer tea.

### 5.5 Regulation of volatile compounds

The aroma and flavor of tea are determined by volatile aroma compounds, which are mainly composed of alcohols, aldehydes, ketones, esters, and terpenes. Several studies reported that applying Se altered the abundance of key aroma compounds in tea leaves, affecting tea aroma profiles (Ye et al., 2023; Xu et al., 2024). Foliar application of nano-Se increased aroma compounds concentration in tea leaves, including ethyl acetate, myrcene, and methyl salicylate (Li et al., 2021). Ye et al. (2023) found that phenylethanol, hexyl acetate, and β-guaiene exhibited a positive correlation with Se concentration of tea leaves, while cis-3-hexenyl hexanoate and indole showed a strong negative correlation with Se. Xu et al. (2024) identified a total of 65 volatile compounds in tea leaves and demonstrated that exogenous GlcN-Se application induced differential regulation of distinct compound groups, which comprehensively improved tea aroma. However, as the application dose of GlcN-Se increased (from 1.67 to 13.33 g L^-1^), the concentrations of aldehydes, ketones, acids, and esters gradually reduced. Conversely, the levels of floral aroma components such as tea furan, dehydrolinalool, nerol, heptanal, and benzaldehyde significantly increased. Notably, tea plants treated with a moderate concentration of GlcN-Se (6.67 g L^-1^) exhibited a greater diversity of volatile compounds and higher total concentrations, indicating that the appropriate dosage of Se optimally improves the concentrations of volatile aroma compounds in tea leaves.

## 6 Conclusion and future perspectives

Numerous studies have indicated that Se-enriched tea possesses greater health benefits than non-enriched tea. Applying the appropriate dose of Se to tea plantations not only promotes tea plant growth but also helps plants tolerate various abiotic stressors. In addition, Se regulates secondary metabolic pathways of tea plants, promoting the synthesis of secondary metabolites that improve tea flavor. Due to the complexity of the soil–plant system in tea gardens, factors such as tea plant varieties, soil properties, and tea processing techniques all influence the Se-enriched tea quality and Se content of tea infusions.

Although exogenous Se application is an efficient method for Se biofortification in tea plantations, long-term Se supplementation may pose potential risks of Se pollution and ecological damage to tea garden soil. Future research on cultivating Se-enriched tea should focus on the following areas: 1) Using molecular breeding techniques to develop Se-rich tea cultivars, reducing exogenous Se application by enhancing the efficiency of Se enrichment; 2) Cultivating Se-enriched tea in native soil by developing soil ameliorants and microbial fertilizers; 3) Enhancing Se bioaccessibility in tea infusions through optimized processing techniques; 4) Investigating the structure and function of new Se-containing small compounds of tea, including Se-containing polyphenols and selenonucleic acids.
